# Evaluation of a Multiplex Electrochemiluminescence Assay for Detection of Anti-Pneumococcal Antibodies in the Diagnosis of Selective Polysaccharide Antibody Deficiency

**DOI:** 10.1007/s10875-025-01911-0

**Published:** 2025-07-10

**Authors:** Nicolas Perrard, Aurore Collet, Sarah Stabler, Sandrine Poizot, Myriam Labalette, Gatien Durand, Frédéric Batteux, Floriane Mirgot, Benjamin Lopez, Sylvain Dubucquoi, Lucie Chevrier, Guillaume Lefèvre

**Affiliations:** 1 Present Address: Department of Internal Medicine and Clinical Immunology, CHU Lille, Lille, F-59000 France; 2 Univ. Lille, Inserm, CHU Lille, U1286 - INFINITE - Institute for Translational Research in Inflammation, Lille, F-59000 France; 3 Institute of Immunology, CHU Lille, Lille, F-59000 France; 4 Regional Center for Primary Immune Deficiency, CEREDIH Lille, CHU Lille, Lille, F-59000 France; 5 Department of Infectious and Tropical Diseases, CHU Lille, Lille, F-59000 France; 6 Univ. Lille, CNRS, Inserm, CHU Lille, Institut Pasteur de Lille, U1019 - UMR 9017 - CIIL - Center for Infection and Immunity of Lille, Lille, F-59000 France; 7 Cochin Hospital, Department of Biological Immunology, Paris, France; 8 Cochin Institute, INSERM U1016, Univ. Paris, Paris, France; 9 Medical Laboratory Department, Dunkerque, Dunkerque, CH France

**Keywords:** Specific antibody deficiency, Selective anti-polysaccharide antibody deficiency, Primary immunodeficiency, Encapsulated bacterial infections, Electrochemiluminescence, ECL

## Abstract

*Streptococcus pneumoniae* can be responsible for severe infections, especially in patients with primary antibody deficiencies like selective anti-polysaccharide antibodies deficiency (SPAD). The reference method recommaned by the World Health Organization for assessment of anti-pneumococcal capsular polysaccharides (PCPs) IgG antibodies is a standardized serotype-specific ELISA (WHO-SSA), but this manual method is time-consuming and limit the number of evaluated PCPs. We aim to evaluate the performance values of a multiplex assay based on electrochemiluminescence (ECL-plex). A panel of 164 sera from 82 patients sampled before and 4–8 weeks after immunization by the 23-valent pneumococcal polysaccharide vaccine (PPV23) were assessed by the reference WHO-SSA (7 to 13 serotypes) and by an 18-plex ECL assay (18 serotypes). All patients had normal serum Ig/subclasses levels and were classified as good (*n* = 43) or poor responders (*n* = 39, i.e. SPAD patients) according to the American Academy of Asthma, Allergy and Immunology’s (AAAAI) current guidelines. We observed excellent correlations between the two methods for anti-PCPs titers against 7 serotypes (*r* = 0.88 [95% CI: 0.87–0.90], *n* = 124 sera) and 13 serotypes (*r* = 0.87 [0.87–0.89], *n* = 40 sera). Using the AAAAI’s guidelines for interpretation, the test performance of the 18-plex ECL assay for SPAD diagnosis showed a sensitivity of 95% and specificity of 84%, positive and negative predictive values of 84% and 95%, respectively. The percentage of agreement was 89% between the SSA and the 18-plex ECL assay. The 18-plex ECL assay is a reliable, rapid, and simple method for evaluating anti-PCPs response and screening for SPAD diagnosis.

**Supplementary Information:**

The online version contains supplementary material available at 10.1007/s10875-025-01911-0.

## Introduction

*Streptococcus pneumoniae* is an encapsulated bacteria responsible for various infections such as otitis, pneumonia, bacteriemia, or meningitis [1, 2]. Unexplained, frequent and/or severe infections might suggest a complement deficiency or antibody deficiency [3]. Pneumococcal capsular polysaccharides (PCPs) allow the identification of more than 100 serotypes [4]. These capsular polysaccharides are the main targets of specific antibodies, enabling opsonization and degradation of the bacteria, while other capsular antigens are immunogenic but do not promote the destruction and clearance of the bacteria [5, 6].

Specific anti-polysaccharide antibody deficiency (SPAD) is characterized by an impaired response to PCPs, leading to recurrent and severe bacterial infections, including pneumococcal infections. This immune deficiency is characterized by normal responses to protein antigens and normal levels of immunoglobulins, T cells, and B cells [7, 8]. The diagnosis must be made according to anti-PCPs response assessed serotype by serotype using a standardized method, both before and 4 to 8 weeks after immunization with the unconjugated 23-valent pneumococcal vaccine (PPV23, PNEUMOVAX^®^) [9]. Interpretation criteria are based on expert consensus regarding specific antibody levels, fold-increases in antibody levels pre/post-immunization and the percentage of good immunization among tested serotypes.

In 2000, a consortium comprising representatives from various academies, governments, and industries, under the auspices of the World Health Organization (WHO), selected a single serotype enzyme-linked immunosorbent assay (ELISA) protocol for quantifying human IgG antibodies specific to PCPs as the reference method [10]. This single-serotype assay (WHO-SSA) is relevant for analyzing serotype-specific antibody levels but is time-consuming and costly in terms of reagents (one plate for each serotype) and sera.

In 2007, Pickering et al. developed an electrochemiluminescence detection assay (ECL-assay) for the multiplex quantification of anti-PCPs antibodies using MesoScale Discovery^®^ technology, with method validation and bridging with WHO-SSA, demonstrating good interchangeability between the two techniques [11]. This assay has been evaluated as a sensitive, time- and serum volume-saving method, and similar performance to WHO-SSA for quantifying serotype-specific anti-PCPs antibodies in pediatric and adult sera [12, 13].

In the present study, we aimed to evaluate ECL-assay performances for diagnosis of SPAD in adults, regarding specific anti-PCPs levels and pre/post-immunization antibody levels using PPV23.

## Materials and Methods

### Polysaccharides

All PCPs powders used for plates (serotypes 1, 3, 4, 5, 6 A, 6B, 7 F, 8, 9 V, 10 A, 11 A, 12 F, 14, 15B, 18 C, 19 A 19 F, 23 F) were courtesy provided by Pfizer^®^. Powders for Cell Wall Polysaccharide (CWPS, Ref 3459) and 22 F (Ref 76966) were provided Statens Serum Institut, Copenhagen, Denmark. In accordance with the product usage recommendations, the powders were reconstituted in type I reagent grade water, with a final concentration following reconstitution at 10 mg/mL. After complete dissolution under agitation at 4 °C using a shaker, the solutions were aliquoted and stored at −80 °C until use for adsorption.

### Calibrant, Sera and External Quality Assessment

We used the International Standard for Human Anti-pneumococcal capsule Reference Serum, lot 007SP as reference serum. The 007sp was prepared from 287 healthy volunteers following vaccination with 23-valent pneumococcal polysaccharide vaccine (PPV23 - PNEUMOVAX II^®^). Calibration for 24 serotype specific IgG concentrations was made in 2011 during an International Collaborative Study involving 5 laboratories [14]. These values were derived following double adsorption of 007sp with cell wall polysaccharide (CWPS) and Ps 22 F [6, 15]. This reference serum is distributed by the U.S. Food and Drug Administration, Bethesda, MD [14, 16].

The concordance between the 18-plex ECL assay and the WHO-SSA was made using sera from 82 adult patients explored before and after vaccination with PPV23, i.e. 164 samples documented in IgG for 7 serotypes (serotypes 4, 6B, 9 V, 14, 18 C, 19 F and 23 F) and of which 40 documented in IgG for 6 more serotypes (7 serotypes plus serotypes 1, 3, 5, 6 A, 7 F and 19 A). All specific anti-PCPs IgG levels were assessed using the WHO-SSA in the immune-monitoring facility at Cochin University Medical Center (Paris, France). These sera were collected from March 2015 to February 2020 [17].

Four samples provided by an external quality assessment service (samples references 19-Dec-2023 236-5/236-6 and 12-Mar-2024 241-5/241-6, UK NEQAS^®^) were tested with the 18-plex ECL. We used the mean results provided after the final analysis of all participating centers (*n* = 6 to 14 depending on the serotype) for comparison with the 18-plex ECL: as it merged results obtained with LUMINEX technology assay and WHO-SSA, this comparison with 18-plex ECL was made independently.

### SPAD Diagnosis

The diagnosis of SPAD was established in patients presenting with unexplained recurrent bacterial infections or at least one severe infection caused by encapsulated bacteria (mostly meningitis and/or pneumonia), in the presence of normal total immunoglobulin levels, normal IgG subclass levels, normal B cell counts, and an impaired anti-polysaccharide antibody response 4 to 8 weeks after immunization with PPV23. A lack of prior PPV23 vaccination within two years before testing was also required to avoid the potential influence of hypo-responsiveness.

The interpretation of anti-PCP antibody responses and the diagnostic criteria for SPAD were defined according to the recommendations of the American Academy of Allergy, Asthma & Immunology (AAAAI). Assessment of pre- and post-immunization anti-PCP antibody titers was performed using the third-generation WHO-SSA [9, 18, 19].

An inadequate response for a given serotype was defined as a post-vaccination antibody concentration below 1.3 mg/L or a failure to achieve a fourfold increase from baseline. A twofold increase was considered acceptable if the baseline level was already above 1.3 mg/L. An overall inadequate response to PCPs was defined as an insufficient response to at least 70% of the tested serotypes (ranging from 7 to 13), leading to the diagnosis of SPAD. The same criteria were applied to the ECL 18-plex assay to assess its diagnostic performance in comparison to the WHO-SSA, which was used as the reference method.

### 18-plex ECL Assay

The MesoScale Discovery^®^ (MSD) technology is based on ECL detection buffer as documented previously [20].

The multispot configuration used two 96-well MSD-multispot carbon microplates with 10 spots/well format. First plate (‘plate A’) was coated with PCPs 1, 3, 4, 5, 6 A, 6B, 7 F, 8 and 9 V, and second plate (‘plate B’) was coated with PCPs 10 A, 11 A, 12 F, 14, 15B, 18 C, 19 A 19 F and 23 F, each at a 100 µg/mL concentration. Each well also contained one CWPS spot 200 µg/mL concentration, which was used to assess and the effectiveness of adsorption with CWPS and 22 F and the background reactivity of the assay.

007sp was used at 8 different 3-fold dilutions (from 1:150 to 1:328,050) to generate a standard curve to determine antibody concentrations in the ECL assays. Sera were tested at a fixed dilution of 1:2,000.

The reference serum 007SP, quality controls, and sera were diluted at appropriate dilutions in phosphate-buffered saline containing 0.05% Tween 20 (PBS-T) and 1% Bovine Serum Albumin (BSA) for blockage, 20 µg/mL CWPS, 20 µg/mL PCPs 22 F for adsorption, and were incubated overnight at 4 °C on a stirring plate. Each antigen-coated plate was incubated at room temperature for 1 h on an orbital shaker at 700 rpm with 5% BSA and PBS-T. Plates were washed 3 times with PBS-T, and 50 µL per well of the preadsorbed and diluted test sera/reference serum/quality control was added and incubated for 2 h at ambient temperature on an orbital shaker. Plates were washed 3 times with PBS-T; and 50µL MSD Sulfo-tag-labeled-goat anti-human IgG secondary antibody diluted 1:1,000 in 1% BSA PBS-T was added to each well and incubated for 1 h at ambient temperature on an orbital shaker. Plates were washed 3 times with PBS-T, and 150 µl of MSD ECL read buffer was added to each well.

**Fig. 1:**
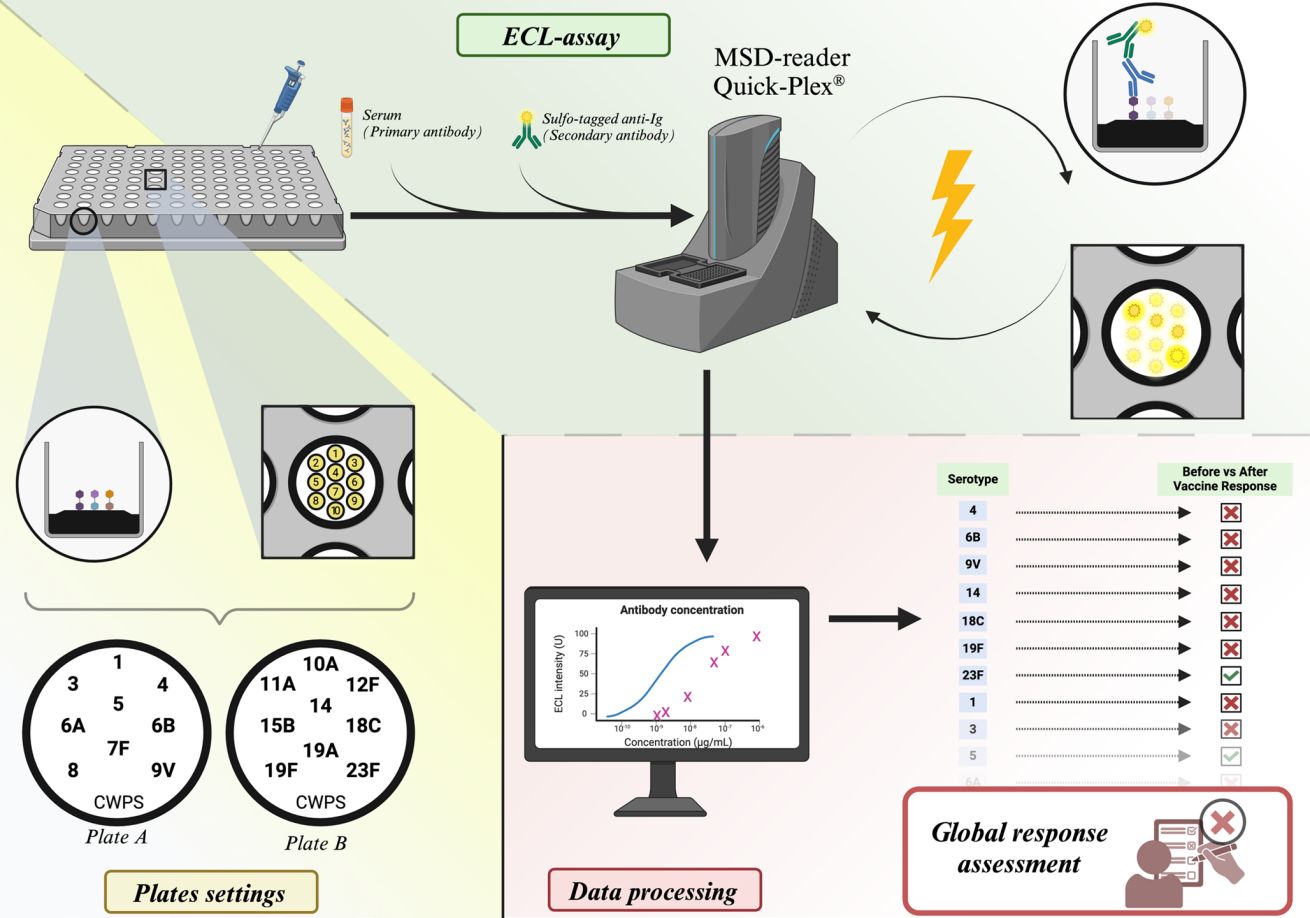
Graphical abstract illustrating the distribution of polysaccharides used on different Meso Scale Discovery^®^* pl*ates, the schematic workflow of the ECL assay, the generation of 4-PL calibration curves based on the 007SP calibrant to determine anti-PCPs concentrations for each serotype, the interpretation of these concentrations for patient global anti-PCPs response and diagnosis of SPAD

Plates were read with the Quick-Plex SQ 120 (MesoScale Discovery^®^). The concentrations of antibodies were determined using MSD Discovery Workbench© software, by referencing their ECL responses against a standard 4-parameter logistic (4PL) model curve generated from the serially diluted 007SP reference serum. This protocol is summarized in Fig. 1.

### WHO-SSA ELISA Method

The WHO-SSA ELISA method used at the Cochin University Medical Center has been previously described and is available online [10]. Briefly, medium-binding 96-well microtiter plates were coated with various concentrations of PCPs including serotypes 1, 3, 5, 4, 6 A, 6B, 7 F, 9 V, 14, 18 C, 19 A, 19 F, and 23 F. Human serum samples and the international reference standard 007SP were pre-adsorbed with 5 µg/mL of CWPS and 5 µg/mL of serotype 22 F polysaccharide for 30 min at room temperature to block antibodies binding to CWPS and other impurities present in pneumococcal polysaccharide coating antigens.

Serial dilutions of the adsorbed sera were then applied to the antigen-coated plates. Serotype-specific antibodies were detected using an alkaline phosphatase-conjugated goat anti-human IgG secondary antibody, followed by the addition of the substrate p-nitrophenyl phosphate. The optical density of each well was measured at 405 nm with a reference at 620 nm using an ELISA plate reader. The optical density values of the samples were compared to those of the standards, and antibody concentrations in human serum were calculated using a 4PL curve-fitting model.

### Statistical Analysis

Correlation between ECL-assay and WHO-SSA was assessed using Spearman correlation test for 7 or 13 serotypes. Concordance between 18-plex ECL and WHO-SSA was assessed using Bland-Altman (difference between each method values versus average) and Deming regression method for 7 and 13 serotypes.

Aberrant values (more than ten times difference between the two methods) were excluded from per-protocol bridging analyses, representing between 1 and 3% of results depending on serotypes, but were kept for SPAD diagnostic performances of the assay.

ROC curves were used to determine the performance of 1.3 µg/mL threshold in ECL-assay compared to WHO-SSA. The combination criteria of 1.3 µg/mL threshold and 2- or 4-times fold change was assessed with percentage of agreement between the two methods, globally and serotype-by-serotype. The level of imbalance in the discordance between 18-plex ECL and UK NEQAS^®^ sera assessment for 0.35 µg/mL threshold, or WHO-SSA for the diagnosis of SPAD was statistically assessed using a McNemar’s exact test. Cohen’s κ-coefficient was also calculated, providing an estimate of the agreement between assays beyond which might exist due to chance alone. The value of the κ-coefficient ranges from − 1.0 to 1.0. Agreement consistent with chance alone yields a κ-coefficient near 0, whereas agreement far exceeding chance alone yields a κ-coefficient approaching 1.0 (from 0.61 to 0.80 being seen as substantial agreement).

Comparisons between pre and post vaccinal response were performed using Mann and Whitney test.

Data analyses and graphs were performed using the GraphPad Prism software version 9.1.2 (GraphPad Software, La Jolla, CA, USA) and online tests provided by developers at https://www.graphpad.com/quickcalcs.

## Results

### 18-plex ECL Set Up

The range and dilutions of calibrant 007SP were visually determined to achieve an optimal 4PL curve at both low and high antibody concentrations for each serotype. We searched for optimal dilutions of patients’ sera and optimal adsorption of non-specific antibodies (see details in Supplementary materials, Supp. Tables 1 and Supp. Figure 1). Briefly, we considered that the optimal measurement range for 007SP started at an initial dilution of 1:150 with seven 1:3 dilutions (final dilution at 1:328,050) that the 1:2,000 dilution of sera had the best signal linearity, and that adsorption of nonspecific antibodies was when sera were incubated overnight (16–24 h) at 2–8 °C with an adsorption solution containing 20 µg/mL of both 22 F and CWPS. The lower and upper limits of detection (LLODs and ULODs) for each polysaccharide at a 1:2,000 dilution are presented in Table 1.

**Table 1:** Detection range for each 18 polysaccharides for 1:2,000 Dilution samples in IgG

IgG (1:2,000 dilution)			IgG (1:2,000 dilution)		
Serotype	LLOD (µg/mL)	ULOD (µg/mL)	Serotype	LLOD (µg/mL)	ULOD (µg/mL)
1	0.097	113.4	10A	0.1134	173
3	0.0208	19.34	11A	0.097	67.8
4	0.0574	44.4	12F	0.0344	29.4
5	0.0608	100.2	14	0.264	506
6A	0.1422	52.4	15B	0.1766	226
6B	0.1918	126.6	18C	0.0624	97.4
7F	0.0824	110.6	19A	0.1186	185
8	0.1448	189.8	19F	0.1234	194.8
9V	0.0716	85.8	23F	0.0772	79.4

*LLOD* Low Lower Limit of Detection, *ULOD* Upper Limit of Detection

### Interassay Comparison

**Fig. 2:**
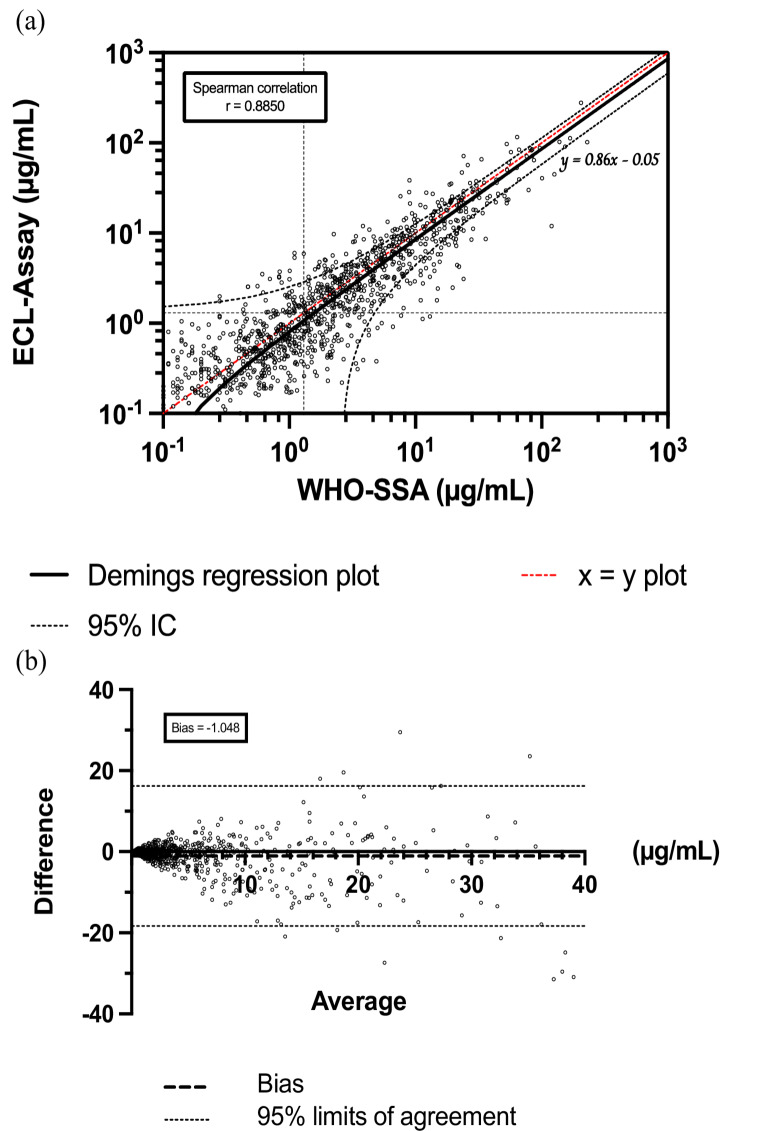
Spearman correlation and Demings’s regression curve between WHO-SSA and 18-plex ECL assay for pooled 7 serotypes *n* = 1,089 (**a**), and Bland Altman analysis illustrating excellent equivalence between WHO-SSA and ECL-assay. Plotted red function represents ideal test x = y

The overall correlation between the 18-plex ECL and WHO-SSA, as determined by Spearman’s correlation test showed excellent correlation across 7 serotypes (164 samples, 1,089 measurements, *r* = 0.8850 [95% CI: 0.8710–0.8975]). Deming correlation showed a regression equation (of the form y = ax + b) of Y = 0.8573*X − 0.05053 for 7 serotypes (slope (a) 95% CI from 0.5939 to 1.121 and Y-intercept (b) 95% CI from − 1.519 to 1.418) revealing an overall trend toward underestimation (Fig. 2a). Bland-Altman tests confirmed a slight tendency for underestimation with the ECL assay for 7 serotypes, with a negative bias of −1.038 and 95% limits of agreement ranging from − 18.31 to 16.21 (Fig. 2b).

This excellent correlation was also found with Spearman correlation across 13 serotypes (164 samples of which 40 were documented for 6 additional serotypes, 1,307 measurements, *r* = 0.8735 [0.8696–0.8852]). Deming regression showed a regression equation of Y = 0.8516*X − 0.08228 for 13 serotypes (slope (a) 95% CI from 0.5983 to 1.105 and Y-intercept (b) 95% CI from − 1.413 to 1.248), while Bland-Altman test showed persistent negative bias of −1.044 and 95% limits of agreement ranging from − 17.09 to 15.00 (Supp. Figure 2).

The serotype-by-serotype assessment showed Spearman’s correlation coefficients ranging from *r* = 0.6693 (0.4241–0.8230) for serotype 6 A to *r* = 0.9626 (0.9215–0.9824) for serotype 19 A. A mild but significative underestimation was observed by the ECL method for serotypes 9 V, 5, and 19 A with Bland-Altman tests and Deming regressions (Supp. Figure 3; Supp Table 2).

**Fig. 3:**
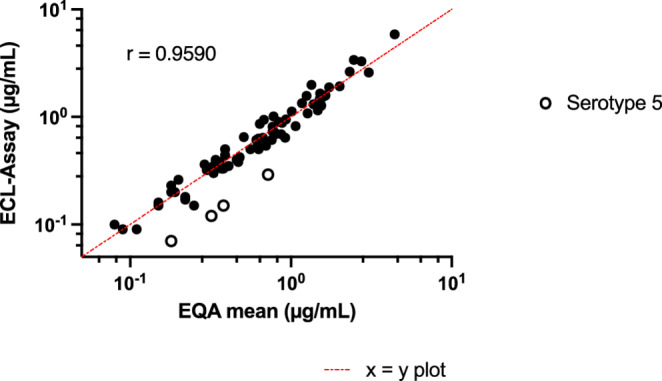
Spearman correlation for 18-plex ECL assay and mean results obtained from 4 samples provided in an external quality assessment (WHO-SSA and Luminex-Based Multiplex Immunoassays) (*n* = 4 for 18 serotypes). Empty dots represented underestimated anti-serotype 5 antibodies. Plotted red function represents ideal test x = y. EQA: External Quality Assessment

There were also excellent correlations with the mean results from the samples collected in an external quality assessment (72 measurements, 4 sera assessed for 18 serotypes each) with Spearman’s correlation coefficients *r* = 0.9590 (0.9342–0.9746) (Fig. 3).

### 18-plex ECL Diagnostic Performance for SPAD

We sought to evaluate the performance of the ECL method for diagnosing SPAD compared to the current gold-standard WHO-SSA.

**Fig. 4:**
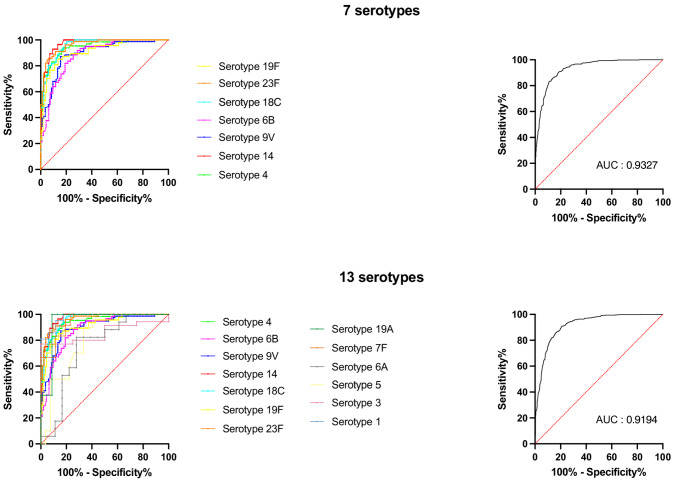
ROC curves for 1.3 µg/mL threshold reliability of the ECL-assay for 7 serotypes (serotypes 4, 6B, 9 V, 14, 18 C, 19 F and 23 F) and 13 serotypes (7 serotypes plus serotypes 1, 3, 5, 6 A, 7 F and 19 A)

Using the threshold defined by expert consensus at 1.3 µg/mL, ROC curves found an AUC of 0.9327 (95% CI: 0.9185–0.9468) across 7 serotypes and 0.9194 (0.9050–0.9338) across 13 serotypes and allows maintaining the threshold of 1.3 µg/mL for the diagnosis of SPAD using the ECL method with a 88.15% sensitivity (95% CI from 85.20 to 90.58%) and 80.40% specificity (95% CI from 77.41 to 83.07%), Youden’s index of 0.69 (Fig. 4).

Using all criteria for SPAD diagnosis (antibody levels, fold increase from pre to post immunization and the number of serotypes with a poor or good response), we observed good diagnostic performances of the 18-plex ECL assay compared to the WHO-SSA, with a sensitivity of 94.9% and specificity of 83.7% (PPV = 84.1%, NPV = 94.7%) in the 82 evaluated patients. The tests did not significantly differ according to McNemar’s exact test (*p* = 0.1824), with a substantial agreement rate (Cohen’s κ-coefficient at 0.781 [95% CI: 0.647 to 0.915], percentage of agreement at 89.02% - Table 2). Among them, 20 patients were diagnosed using a 13-serotype evaluation and 62 with a 7-serotype evaluation. When focusing on the 20 patients diagnosed using a 13-serotype evaluation, the diagnostic performance increased to a sensitivity of 100% and specificity of 85.7% (PPV = 92.9%, NPV = 100% - Supp. Table 3).

**Table 2:** SPAD diagnostic performance of 18-plex ECL-assay compared with WHO-SSA: global performancen = 82 (controls *n* = 43, patients with SPAD *n* = 39)

18-plex ECL-assay *N* = 82	WHO-SSA Global performance		
	*SPAD +*	*SPAD -*	
*Test +*	*37*	*7*	***PPV: 84.1%***
*Test -*	*2*	*36*	***NPV: 94.7%***
	***Se: 94.9%***	***Sp: 83.7%***	
	*Cohen’s κ-coefficient*:		*0.781*
	*% of agreement:*		*89.02%*
	*McNemar’s exact p-value*:		*0.1824*

*N/PPV *Negative/Positive Predictive Value,* Se *Sensibility,Sp* Specificity*,SSA* Single Serotype Assay*

### Illustration of anti-PCPs Antibody Heterogeneity

**Fig. 5:**
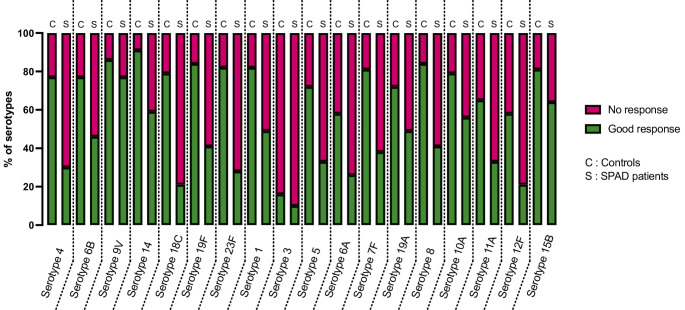
Assessment of specific responses to PCPs in the 18-plex ECLA assay in SPAD patients and controls grouped according to serotype. For each serotype, a good response was considered according to the AAAAI’s composite criteria taking account of pre- and post-PPV23 antibody levels. A “good response” was defined if (i) the post-immunization anti-body titer was upper 1.3 µg/mL and (ii) it achieved a 4-fold increase relative to the pre-immunization value or achieved a 2-fold increase if the pre- immunization value was already greater than 1.3 µg/mL. If one of the 2 criteria was absent, it was considered as a “poor response”

Using the results of the ECL-assay, serotype-by-serotype response (combined 1.3 µg/mL threshold and 2- or 4-times fold change depending on prevaccinal rate) were assessed across 18 serotypes for 43 controls and 39 patients with SPAD revealing variability among serotypes. Specifically, a low response rate (< 20%) was observed for serotype 3, whereas an excellent response rate (> 80%) was noted for serotype 9 V in both SPAD patients and controls. The most discriminating serotype for SPAD diagnosis appears to be serotype 18 C, for which a good response was observed in 79% of control subjects compared to only 21% of SPAD patients (Fig. 5). The median response for all serotypes was significantly different between controls and SPAD patients (79.0% [IQR 70.25-91.0%] vs. 39.5% [IQR 27.5-50.75%], respectively, *p* < 0.0001 - Supp. Figure 4).

## Discussion

We introduce a new method for assessment of anti-PCPs antibody response to PPV23 in the diagnosis of primary antibody deficiencies. Our results suggest that the 18-plex ECL assay can be considered to diagnose SPAD and compensate for the disadvantages of WHO-SSA.

Our results demonstrate excellent correlation with the WHO-SSA reference method for measuring specific anti-PCPs antibody levels using ELISA method despite a tendency to underestimate antibody concentrations. This strong correlation has been previously demonstrated by several research teams [11–13, 20, 21].

The ECL-assay performs well in screening for SPAD with excellent sensitivity and NPV. Due to the heterogeneous response among anti-PCPs responses, diagnosing SPAD could be more reliable on more than 7 serotypes. This heterogeneity could also partly explain the discrepancies observed with the ECL-assay on 13 and 18 polysaccharides. By comparing the response rate to each polysaccharide and the previous work from our team [17], a consistent difference is observed in most serotypes between SPAD patients and controls, suggesting that each explored polysaccharide could potentially improve the performance of SPAD diagnosis.

However, it appears that specific serotypes may be underestimated by the 18-plex ECL assay (for example serotype 9 V, 5, and 19 A) or exhibit weaker correlation with the WHO-SSA compared to others. This last point is particularly notable for serotypes 6 A and 6B, which could potentially be affected by cross-reactivity within the same serogroup [4, 22]. Some serotypes also appear to be more (for example serotype 18 C) or less (serotype 3 and 9 V) discriminating for SPAD diagnosis, although the diagnostic criteria do not specify which serotypes should be tested specifically. Subsequent advancements are imperative to ascertain the optimal coating concentration of PCPs within the well, which PCPs and their distribution across plates, to mitigate methodological interference and cross-reactivity between serogroups.

More in-depth explorations of the pathogenesis of SPAD seem necessary in order to ultimately redefine the diagnostic criteria for this rare immune deficiency [23].

## Conclusion

The ECL is a promising method for exploring anti-PCPs immunity and diagnosing SPAD. We demonstrate an excellent reproducibility of results and diagnostic performance compared to the WHO-SSA reference method.

## Electronic Supplementary Material

Below is the link to the electronic supplementary material.

Supplementary Material 1 (DOCX 1.22 MB)

## Data Availability

No datasets were generated or analysed during the current study.

## Abbreviations

4PL: 4-parameter logistic curve
AAAAI: American Academy of Allergy, Asthma & Immunology
BSA: Bovine Serum Albumin
CV: Bovine Serum Albumin
CWPS: Cell Wall Polysaccharides
ECL-assay: Electrochemiluminescence Detection Assay
ELISA: Enzyme-linked immunosorbent assay
EQA: External quality assessment
IQR: External quality assessment
MSD: MesoScale Discovery^®^
N/PPV: Negative/Positive Predictive Value
OPA: Opsonophagocytosis Assay
PBS: Phosphate-buffered Saline
PCPs: Pneumococcal Capsular Polysaccharide
PPV23: Pneumococcal Polysacharide Vaccine 23-valent
SPAD: Specific anti-polysaccharide antibody deficiency
U/LLOD: Upper/Lower Limit Of Detection
WHO-SSA: World Health Organization-Single Serotype Assay
