# Supplementary material for: Evaluation of a Multiplex Electrochemiluminescence Assay for Detection of Anti-Pneumococcal Antibodies in the Diagnosis of Selective Polysaccharide Antibody Deficiency
Source: J Clin Immunol. 2025 Jul 10;45(1):112. doi: 10.1007/s10875-025-01911-0 (PMC12245944; doi:10.1007/s10875-025-01911-0)
Supplement: Supplementary file 1 — Supplementary Material 1 (DOCX 1.22 MB) [file 10875_2025_1911_MOESM1_ESM.docx]

**Supplementary appendix**

**Protocol optimization**

The range and dilutions of calibrant 007SP were visually determined to achieve an optimal 4PL curve at both low and high antibody concentrations for each serotype. The optimal measurement range for 007SP started at an initial dilution of 1:150 with seven 1:3 dilutions (final dilution at 1:328,050). This dilution allowed for the use of minimal serum (10µL per sample), achieving the optimal ECL Lower Limit of Detection (LLOD) and Upper Limit of Detection (ULOD) for pre- and post-vaccination levels to support interpretation of threshold of 1.3 mg/L and a 2- to 4-fold-change in vaccine response. These ULOD and LLOD were automatically determined by the MSD Discovery Workbench© software based on the parameters of the 4-PL calibration curve equation. As an example, the highest ULOD and LLOD were obtained for serotype 14 due to its high concentration of anti-PCPs antibodies in 007SP, although this detection range still allows for the interpretation of the vaccine response for this serotype. (Supp. Figure 1 - see main text [14]).

Twelve patient samples were initially tested at two serial dilutions of 1:500, 1:1,000 and 1:2,000, the 1:2,000 dilution had the best signal linearity and was chosen for the inter assay comparison.

Adsorption of non-specific antibodies by 22F and CWPS, was compared between 10 and 20 µg/mL each in the adsorption solution: 20 µg/mL was optimal to negate the CWPS spot signal at a dilution of 1:2,000. For the same serum, anti-PnPS levels measured using the ECL method were consistently lower when adsorption was performed overnight at 4°C with agitation compared to one hour at room temperature. This finding indicates more effective adsorption of non-specific anti-PnPS antibodies with the overnight protocol.

Protocol optimization is summarized in Supp. Table 1.

**Figures**


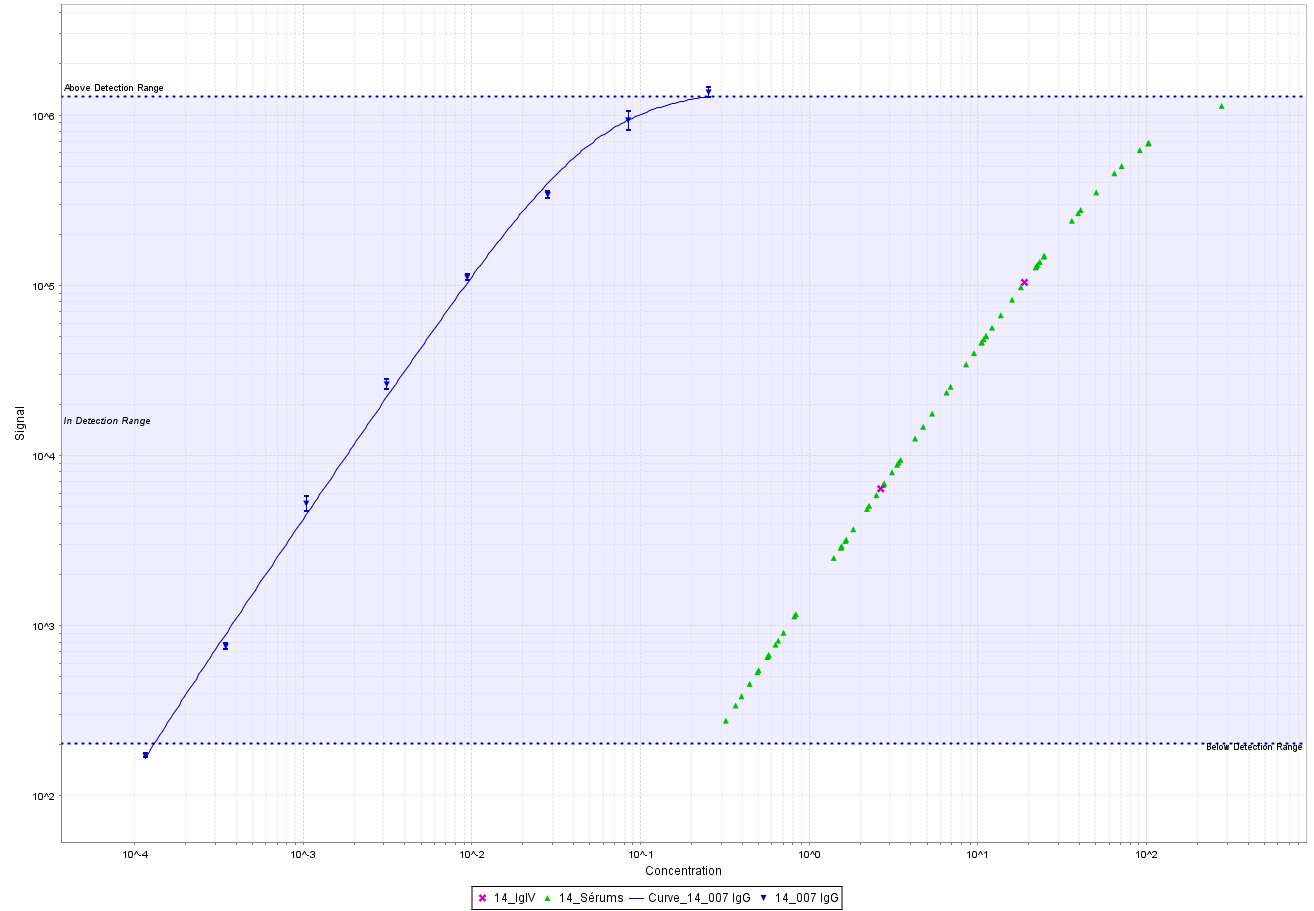


*Supp. Figure 1: Extraction of 007SP serotype 14 4-PL calibration curve from MSD Discovery Workbench© in a concentration(µg/mL)/signal graphic (8 points in duplicates, blue triangles and curve), with illustration of samples determination of anti-PnPS serotype 14 antibody (green triangles).*

*(a)*

**

*(b)”*

**

*Supp figure 2: Spearman correlation and Demings’s regression curve between WHO-SSA and 18-plex ECL assay for pooled 13 serotypes n = 1,307 (a), and Bland Altman analysis illustrating excellent equivalence between WHO-SSA and ECL-assay. Plotted red function represents ideal test x = y.*

*Supp. Figure 3:* *Detailed Spearman correlation (a) and Bland-Altman tests (b) for serotypes 4, 6B, 9V, 14, 18C, 19F and 23F (n = 154 to 158 per serotype), and for serotypes 1, 3, 5, 6A, 7F and 19A (n = 35 to 39 per serotype)*

*Supp. figure 4: Assessment of specific responses to PCPs in the 18-plex ECLA assay in SPAD patients and controls, grouped according to diagnosis.*

**Tables**

| *Supp. Table 1: Parameters tested in the ECL-assay* | | |
| --- | --- | --- |
| **Assessed parameter** | Conditions | Retained conditions |
| **007 standard curve** | From 1:150 to 1:364,500  1:2 or 1:3 serial dilutions  7 or 8 points in duplicats | 1:500 to 1:328,050  1:3 serial dilutions  8 points in duplicats |
| **Sample dilution** | From 1:500 to 1:2,000 | 1:2,000 |
| **Adsorbants quantity** | 10 or 20 μg/mL | 20 μg/mL |
| **Adsorptions duration** | 1 hour RT or O/N 4°C | O/N 4°C |
| *RT: room temperature, O/N: over night* | | |

*Supp Table 2: Detailed Bland-Altman tests bias and agreement with standard deviation of bias and 95% limits of agreement for 7, 13 and individual serotypes (a), and Deming regression equations (type y = ax+b) with 95% Confidence Intervals for slope and Y intercept for 7, 13 and individual serotypes (b)*

(a)

| **Serotype** | **Biais (Standard deviation)** | **95% LOA** |
| --- | --- | --- |
| **4**  **(n=155)** | -0.6205 (SD = 2.013) | -4.567 to 3.325 |
| **6B**  **(n=154)** | -0.5709 (SD = 8.452) | -17.14 to 15.99 |
| **9V**  **(n=158)** | -1.114 (SD = 3.686) | -8.339 to 6.110 |
| **14**  **(n=155)** | -2.285 (SD = 16.06) | -33.77 to 29.19 |
| **18C**  **(n=154)** | -0.2045 (SD = 5.880) | -11.73 to 11.32 |
| **19F**  **(n=157)** | -1.793 (SD = 11.85) | -25.02 to 21.43 |
| **23F**  **(n=156)** | -0.7320 (SD = 4.600) | -9.747 to 8.283 |
| **Total 7 serotypes**  **(n=1089)** | -1.048 (SD = 8.807) | -18.31 to 16.21 |
| **1**  **(n=38)** | -1.045 (SD = 3.873) | -8.636 to 6.547 |
| **3**  **(n=39)** | 0.07144 (SD = 0.8838) | -1.661 to 1.804 |
| **5**  **(n=37)** | -2.771 (SD = 3.331) | -9.301 to 3.758 |
| **6A**  **(n=35)** | 0.5302 (SD = 2.760) | -4.880 to 5.941 |
| **7F**  **(n=38)** | -1.582 (SD = 3.250) | -7.953 to 4.788 |
| **Total 13 serotypes**  **(n=38)** | -1.044 (SD = 8.187) | -17.09 to 15.00 |

(b)

| **Serotype** | **Demings regression equation**  **(y = ax + b)** | **Slope (a) 95% Confidence Intervals** | **Y intercept (b) 95% Confidence Intervals** | **Is slope significantly non-zero?** |
| --- | --- | --- | --- | --- |
| **4**  **(n=155)** | Y = 0.6509*X + 0.1742 | 0.4179 to 0.8839 | -0.1860 to 0.5344 | Significant  <0.0001 |
| **6B**  **(n=154)** | Y = 0.7325*X + 0.9617 | 0.01616 to 1.449 | -1.933 to 3.856 | Significant  <0.0001 |
| **9V**  **(n=158)** | Y = 0.6240*X + 0.3328 | 0.4474 to 0.8006 | -0.04505 to 0.7106 | Significant  <0.0001 |
| **14**  **(n=155)** | Y = 0.8790*X + 0.05941 | 0.4815 to 1.276 | -5.241 to 5.359 | Significant  <0.0001 |
| **18C**  **(n=154)** | Y = 1.226*X - 1.357 | 0.5124 to 1.939 | -4.082 to 1.368 | Significant  <0.0001 |
| **19F**  **(n=157)** | Y = 0.6023*X + 1.399 | -0.03136 to 1.236 | -2.206 to 5.005 | Significant  <0.0001 |
| **23F**  **(n=156)** | Y = 0.8721*X - 0.1416 | 0.4260 to 1.318 | -1.578 to 1.295 | Significant  <0.0001 |
| **Total 7 serotypes**  **(n=1089)** | Y = 0.8573*X - 0.05053 | 0.5939 to 1.121 | -1.519 to 1.418 | Significant  <0.0001 |
| **1**  **(n=38)** | Y = 0.7112*X + 0.2272 | 0.3123 to 1.110 | -0.6279 to 1.082 | Significant  <0.0001 |
| **3**  **(n=39)** | Y = 0.9545*X + 0.1019 | -0.8351 to 2.744 | -0.7380 to 0.9418 | Significant  <0.0001 |
| **5**  **(n=37)** | Y = 0.4465*X - 0.3390 | 0.3781 to 0.5150 | -0.6440 to -0.03402 | Significant  <0.0001 |
| **6A**  **(n=35)** | Y = 1.440*X - 0.6408 | 0.9338 to 1.946 | -1.371 to 0.08888 | Significant  <0.0001 |
| **7F**  **(n=38)** | Y = 0.7520*X - 0.4393 | 0.2488 to 1.255 | -1.832 to 0.9538 | Significant  <0.0001 |
| **19A**  **(n=31)** | Y = 0.5543*X + 1.989 | 0.2984 to 0.8102 | 0.6935 to 3.284 | Significant  <0.0001 |
| **Total 13 serotypes**  **(n=38)** | Y = 0.8516*X - 0.08228 | 0.5983 to 1.105 | -1.413 to 1.248 | Significant  <0.0001 |

| *Supp. Table 3: SPAD diagnostic performance of 18-plex ECL-assay compared with WHO-SSA : 7 or 13 serotype performance* | | | |
| --- | --- | --- | --- |
| ***ECL-assay***  *N=62* | ***WHO-SSA***  *7 serotype* | | |
|  | *SPAD +* | *SPAD -* |  |
| *Test +* | *24* | *6* | ***PPV: 80.0%*** |
| *Test -* | *2* | *30* | ***NPV: 93.8%*** |
|  | ***Se: 92.3%*** | ***Sp: 83.3%*** |  |
| ***ECL-assay***  *N=20* | ***WHO-SSA***  *13 serotype* | | |
|  | *SPAD +* | *SPAD -* |  |
| *Test +* | *13* | *1* | ***PPV: 92.9%*** |
| *Test -* | *0* | *6* | ***NPV: 100%*** |
|  | ***Se: 100%*** | ***Sp: 85.7%*** |  |
| *N/PPV: Negative/Positive Predictive Value, Se: Sensibility, Sp: Specificity, SSA: Single Serotype Assay* | | | |
